# Transcatheter Intracerebral Laser Photobiomodulation Therapy ‐ Important Direction in Alzheimer's Disease Treatment Long‐Term Results

**DOI:** 10.1002/alz70855_101562

**Published:** 2025-12-23

**Authors:** Ivan V Maksimovich

**Affiliations:** ^1^ Clinic of Cardiovascular Diseases named after Most Holy John Tobolsky, Moscow, Moscow, Russia

## Abstract

**Background:**

Improving cerebral microcirculation, reducing cerebral atrophy and dementia severity are important directions in AD treatment.

We present long‐term results of Transcatheter Intracerebral Laser Photobiomodulation Therapy in AD treatment.

**Method:**

The research included 48 patients previous diagnosed with AD. The examination included: CDR, TDR, MMSE assessment, cerebral MRI, MRA, CT, MSCTA, scintigraphy (SG), rheoencephalography (REG), cerebral multi‐gated angiography (MUGA).

According to dementia severity, patients were divided into: preclinical stage TDR‐0 ‐ 4 patients, mild stage TDR‐1 ‐ 16, moderately severe stage TDR‐2 ‐ 21, severe stage TDR‐3 ‐ 7.

All patients underwent Transcatheter Intracerebral Laser Photobiomodulation Therapy (PBMT).

**Result:**

In the long‐term period (1‐10 years), due to stimulation of cerebral angiogenesis and neurogenesis using Transcatheter Intracerebral Laser Photobiomodulation Therapy (PBMT), in all 48 (100%) cases, cerebral blood supply and microcirculation improved, cerebral involutional and atrophic changes decreased.

Only 1 year after PBMT, all 4 (100%) patients with preclinical AD stage TDR‐0 showed complete restoration of temporal lobes volume, no signs of dementia or cognitive impairment; positive dynamics remained over the ten‐year observation period.

All 16 (100%) patients with mild AD stage TDR‐1 showed temporal lobes volume 8‐15.5% increase, no signs of dementia or cognitive impairment; positive dynamics persisted over the ten‐year observation period.

In patients with moderately severe AD stage TDR‐2, temporal lobes volume increased by 5‐15%, dementia decreased, cognitive functions improved. Positive dynamics maintained for 4‐4.5 years.

In patients with severe AD stage TDR‐3, temporal lobes volume increased by 6‐12%, dementia decreased, cognitive functions improved. Positive dynamics maintained for 2‐2.5 years.

The overwhelming majority of patients were transferred to milder AD stage groups.

**Conclusion:**

Transcatheter Intracerebral Laser Photobiomodulation Therapy (PBMT) is an effective, physiologically based method for stimulating cerebral angiogenesis and neurogenesis in AD patients. As a result of complex laser exposure, patients experience cerebral collateral and capillary revascularization, tissue metabolism improves, and cerebral regenerative processes develop. Clinically, this leads to a persistent decrease in dementia level and cognitive functions restoration. After the treatment, the clinical effect lasts for a long time.

